# Supplementary material for: Comparison of Swim-Up and Microfluidic Sperm Sorting Methods in Selection of Sperm for Intracytoplasmic Sperm Injection
Source: Int J Mol Sci. 2025 Jun 4;26(11):5374. doi: 10.3390/ijms26115374 (PMC12155474; doi:10.3390/ijms26115374)
Supplement: Supplementary file 1 [file ijms-26-05374-s001.zip › Table S1.pdf]

Table S1: Separation efficiency in patients with a concentration lower than 10 mil/ml

This table only included patients with an initial concentration lower than 10 mil/ml. Here are the results obtained after separation by the swim-up and MFSS methods.

Sample	Neat		Evaluation	Swim-up		MFFS	
	c (mil/mL)	tsc		c (mil/mL)	tsc (mil)	c (mil/mL)	tsc (mil)
250255	6	18	O	0,4	0,194	0,2	0,097
240797	5	18	OAT	0,5	0,125	0,4	0,16
240784	7	26,6	OAT	0,8	0,32	0,5	0,2375
240743	8	22,4	O	1	0,45	1	0,475
240727	9	34,2	O	1,5	0,675	2,6	1,261
240726	2	5,2	OAT	0,1	0,025	0,1	0,0475
240667	7	22,4	OAT	0,6	0,285	0,1	0,0475
240646	6	24	OAT	0,3	0,135	0,2	0,09
240614	6	25,4	OT	0,8	0,388	0,4	0,19

O –oligozoospermia, OAT – oligoasthenozoospermia, OT – oligoteratozoospermia

c – concentration, tsc – total sperm count

For the optimal technique, a minimum volume of 0.1 ml/ml after separation was addressed, and only one patient had less than 0.1 mil/ml after swim-up, while there were four patients with MFFS (SupplS3).
