# Supplementary material for: Comparison of Swim-Up and Microfluidic Sperm Sorting Methods in Selection of Sperm for Intracytoplasmic Sperm Injection
Source: Int J Mol Sci. 2025 Jun 4;26(11):5374. doi: 10.3390/ijms26115374 (PMC12155474; doi:10.3390/ijms26115374)
Supplement: Supplementary file 1 [file ijms-26-05374-s001.zip › Table S2.pdf]

Table S2: Comparison of the stability of ROS/RNS in ejaculate before their spermatozoa separation.

Samples were frozen at three time intervals after ejaculation. First, samples were collected immediately after transport to the laboratory (within 5 min of ejaculation - 0 min time for liquefaction in the laboratory). The second group were samples that that were at room temperature for 60 min after transport to the laboratory (the standard liquefaction time routinely used before ejaculate examination). The third group were samples that were at room temperature for 180 min after ejaculation.

Patient no.	0 min	60 min	180 min
1	100%	70%	73%
2	100%	71%	74%
3	100%	61%	57%
4	100%	92%	79%
Average	100%	73,5%	70,75%

Table show the relative comparison of the decrease in ROS/RNS levels over time. From this table it is clear that during the first 60 min, the ROS/RNS levels in the sample decrease approximately 73.5 % of the original values. The further decrease at RT is not as dramatic and the values decrease linearly so that after 180 minutes they are on average at 70.75% of the original values.
