# Supplementary material for: Comparison of Swim-Up and Microfluidic Sperm Sorting Methods in Selection of Sperm for Intracytoplasmic Sperm Injection
Source: Int J Mol Sci. 2025 Jun 4;26(11):5374. doi: 10.3390/ijms26115374 (PMC12155474; doi:10.3390/ijms26115374)
Supplement: Supplementary file 1 [file ijms-26-05374-s001.zip › Table S3.pdf]

Table S3: Stability of ROS/RNS values during storage

Patients	Time of storage (months)	ROS/RNS concentration [eq. µM H_2O_2]		% TO
		-20 °C	-196 °C	
1	36	5,02	84,20	94
2	36	2,73	120,20	97,7
3	24	1,47	59,27	97,5
4	24	0,80	58,65	98,63
5	12	0,17	86,95	99,8
6	12	1,22	57,49	97,87
7	6	2,69	37,37	92,8
8	6	1,00	78,08	98,7
9	1	0,32	71,92	99,55
10	1	6,75	82,29	91,79
Average		2,22[a]	73,64[b]	96,83

Different indexes (a.b) shows statisticaly significant differences (ANOVA, $p<0,001$).

Verification of ROS/RNS stability in frozen samples during storage in liquid nitrogen (-196 °C) and at -20 °C. Both short-term storage (1 month) and longer time intervals (6, 12, 24, 36 months) were monitored. During storage, there was a dramatic decrease in ROS/RNS concentration in ejaculate stored at -20 °C by more than 95% of the value compared to samples stored in liquid nitrogen.
